# Detection-Guided ROI-Constrained Diffusion for Weakly Supervised White Blood Cell Segmentation: A Retrospective Internal and External Dataset Evaluation

**DOI:** 10.3390/jcm15176846

**Published:** 2026-09-03

**Authors:** Julius Bamwenda, Mehmet Siraç Özerdem, Orhan Ayyildiz, Veysi Akpolat, İrem Akpolat

**Affiliations:** 1 Electrical & Electronics Engineering Department, Engineering Faculty, Dicle University, 21280 Diyarbakır, Türkiye; hbamwenda@gmail.com (J.B.); sozerdem@dicle.edu.tr (M.S.Ö.); 2 Department of Internal Medicine-Hematology, Medical Faculty, Dicle University, 21280 Diyarbakır, Türkiye; orhanay21@hotmail.com; 3 Department of Biophysics, Medical Faculty, Dicle University, 21280 Diyarbakır, Türkiye; 4 Department of Nutrition and Dietetics, İstanbul Okan University, 34959 İstanbul, Türkiye; iremakpolat7@gmail.com

**Keywords:** white blood cells, weak supervision, pseudo-masks, YOLOv13, diffusion model, cell segmentation, ROI-constrained segmentation, external validation

## Abstract

**Background**: Accurate white blood cell (WBC) segmentation is important for quantitative microscopic image analysis, but conventional supervised approaches depend on labor-intensive pixel-level annotations and may exhibit reduced robustness across datasets. This study proposes a detector-guided, region of interest (ROI)-constrained framework that combines automatic localization, pseudo-mask-based weak supervision, and diffusion-assisted segmentation. **Methods**: You Only Look Once version 13 Nano (YOLOv13-N) was used to localize WBCs and define ROIs, within which segmentation was performed using the proposed diffusion-assisted model. Automatically generated pseudo-masks served as segmentation-training targets, while expert masks were retained for reference evaluation. Experiments used a verified Dicle cohort of 14,721 records, partitioned into 10,305 training, 2208 validation, and 2208 held-out test records. Performance was evaluated separately at ROI-conditional and end-to-end levels. Generalization was assessed on 11,200 Raabin-WBC records using the frozen framework without external tuning. **Results**: The automatically generated pseudo-masks achieved a mean Dice score of approximately 0.702, demonstrating usable but imperfect weak supervision. On the held-out Dicle test set, the proposed framework achieved a Dice score of 0.7188, intersection over union (IoU) of 0.5796, precision of 0.8966, and recall of 0.6422 under ROI-conditional evaluation. End-to-end performance was 0.6705 Dice, 0.5408 IoU, 0.8378 precision, and 0.5986 recall, demonstrating the influence of localization on overall performance. Comparison with reference segmentation architectures showed that the proposed framework did not maximize internal Dice, while achieving the highest reported ROI-conditional precision. Frozen external evaluation on Raabin-WBC revealed further degradation under dataset shift, with failure analysis identifying detector/ROI transfer as an important end-to-end bottleneck. **Conclusions**: The proposed framework demonstrates the feasibility of WBC segmentation using detector-guided ROI processing and pseudo-mask-based weak supervision, reducing reliance on expert pixel-level segmentation targets. The findings further show that robust cross-dataset localization is critical to end-to-end generalization and provide a clear direction for improving weakly supervised WBC segmentation across heterogeneous microscopy datasets.

## 1. Introduction

Microscopic examination of peripheral blood smears remains an important component of hematological assessment. The appearance of white blood cells (WBCs), including their size, shape, nuclear structure, cytoplasmic characteristics, and staining patterns, provides useful information for cell characterization and subsequent clinical interpretation. In computational microscopy, accurate segmentation is required to separate WBCs from surrounding erythrocytes, platelets, and background regions and to obtain reliable measurements of cellular and nuclear morphology. Recent studies have therefore investigated automated approaches for WBC detection, classification, and segmentation to support quantitative blood-smear analysis [1,2]. Large-scale resources such as Raabin-WBC have also contributed to the development and evaluation of automated WBC analysis methods by providing heterogeneous microscopic images acquired under different imaging conditions [3]. Nevertheless, reliable WBC segmentation remains challenging because variations in staining, illumination, acquisition conditions, cell morphology, and background composition can substantially alter image appearance. Cells may also touch or overlap, while weak contrast and irregular cellular boundaries can complicate accurate delineation [1,3].

Deep learning has substantially advanced biomedical image segmentation. U-Net introduced the widely adopted encoder–decoder architecture with skip connections and remains a fundamental reference for biomedical segmentation [4]. Subsequent architectures extended this principle by improving feature fusion, attention, and adaptation to individual datasets. Attention U-Net introduced attention gates that allow the network to emphasize task-relevant structures [5], while UNet++ redesigned encoder–decoder connections through nested dense skip pathways to reduce the semantic gap between feature levels [6]. nnU-Net subsequently demonstrated that systematic adaptation of preprocessing, network configuration, training, and post-processing to dataset characteristics can produce strong performance across diverse biomedical segmentation tasks [7]. These developments have established convolutional neural networks (CNNs) as strong segmentation baselines. However, their performance typically depends on sufficiently large sets of accurately annotated training images, and obtaining expert pixel-level masks remains costly and time-consuming in medical imaging [7,8,9]. The annotation burden becomes particularly relevant for microscopy studies containing thousands of individual cells.

Transformer-based architectures have further expanded the design space for medical image segmentation by enabling explicit modeling of long-range spatial relationships. TransUNet combines convolutional feature extraction with transformer-based contextual modeling [10], whereas Swin-Unet employs hierarchical shifted-window transformers within a U-shaped architecture [11]. UNETR similarly demonstrated the use of transformer encoders coupled with U-shaped decoding for medical segmentation [12]. These approaches can capture broader contextual relationships than purely local convolutional operations, but they do not inherently eliminate the need for accurately annotated training data. Consequently, annotation-efficient learning remains an important consideration even as segmentation architectures become increasingly sophisticated [9,10,11,12].

More recently, foundation models have introduced a prompt-driven paradigm for image segmentation. The Segment Anything Model (SAM) demonstrated that a large segmentation model trained on diverse natural images can generate object masks from prompts such as points and bounding boxes [13]. However, direct transfer from natural-image pretraining to medical images is not consistently reliable because of substantial domain differences in image appearance, scale, and target structure [14,15,16]. This observation has motivated medical adaptations of SAM. MedSAM, for example, was developed using a large collection of medical image–mask pairs spanning multiple imaging modalities [17], while SAM-Med2D explored extensive medical-domain fine-tuning for two-dimensional segmentation [18]. Medical SAM Adapter introduced parameter-efficient adaptation mechanisms for incorporating medical-domain information into SAM [19]. These developments demonstrate the potential of foundation models while also emphasizing that domain adaptation and appropriate prompting remain important for medical segmentation.

SAM 2 extended promptable segmentation toward unified image and video processing [20], and subsequent studies have investigated its applicability to medical imaging and video data [21]. Other approaches have combined localization with foundation-model segmentation; MedLSAM, for example, incorporates localization information to facilitate segmentation of medical targets [22]. Such developments are particularly relevant to microscopy because a localization stage can provide spatial guidance before detailed boundary delineation. Nevertheless, promptable foundation models do not by themselves guarantee a fully automated solution for every medical segmentation task, particularly when reliable prompt generation and domain transfer must also be addressed [16,17].

Diffusion models provide a different approach to segmentation. Denoising diffusion probabilistic models learn a reverse process that progressively recovers structured information from noisy representations [23]. Although initially developed primarily for generative modeling, diffusion methods have subsequently been investigated across medical imaging applications, including reconstruction, synthesis, anomaly detection, and segmentation [24]. MedSegDiff adapted diffusion probabilistic modeling specifically to medical image segmentation using dynamic conditional encoding and frequency-aware feature processing [25], while MedSegDiff-V2 incorporated transformer mechanisms into a diffusion-based segmentation framework and evaluated the approach across multiple medical segmentation tasks [26]. These studies demonstrate the potential of diffusion-based segmentation, but iterative denoising and sampling can introduce greater computational demands than conventional single-pass segmentation networks [24,25,26].

One strategy for limiting unnecessary image processing is to localize a candidate cell before performing detailed segmentation. Detection-first approaches have already been explored for WBC analysis, including systems that combine YOLO-based localization with subsequent classification or feature analysis [2]. In such frameworks, the detector identifies the spatial region containing the target cell, allowing the subsequent segmentation model to operate on a smaller and more relevant portion of the image. This strategy is particularly suitable for peripheral blood-smear microscopy, where a target WBC may occupy only a fraction of the available field. Restricting segmentation to a detector-derived region of interest (ROI) can therefore reduce exposure to irrelevant background information and establish a consistent spatial input for downstream segmentation [1,2]. Importantly, however, a sequential detection–segmentation architecture also introduces dependency between stages: missed or inaccurate localization can propagate directly to the final segmentation result.

A second challenge is the dependence on detailed manual segmentation masks. Weakly and semi-supervised learning strategies seek to reduce annotation requirements by exploiting incomplete, coarse, noisy, or automatically generated supervisory information [8,9]. This is particularly relevant to medical imaging, where reliable pixel-level annotations often require substantial expert effort. Recent weakly supervised segmentation approaches have shown that useful pixel-level representations can be learned from less complete forms of supervision and pseudo-labeling strategies [27,28]. The broader literature on learning with limited annotations similarly demonstrates the potential of combining restricted labeled data with automatically derived supervisory information [9]. Weak supervision should, however, be distinguished from unsupervised learning: pseudo-masks or approximate annotations still provide an explicit training signal even when they are not manually delineated reference masks [8].

These developments suggest an opportunity to combine automatic localization, restricted ROI processing, and weak segmentation supervision within a single framework. Detection can spatially constrain the segmentation problem, while automatically generated pseudo-masks can reduce reliance on manual pixel-level training annotations. Diffusion-based segmentation can then be applied within the localized region rather than across the entire microscopy image. Such a formulation also permits localization and segmentation to be evaluated separately, which is important because strong segmentation performance within correctly localized ROIs does not necessarily imply equivalent end-to-end performance when detector failures are included.

The present work is also related to our recently published TransNet–SAM2 study, which investigated prompt-free WBC segmentation using transformer-based feature extraction together with a SAM2 segmentation framework [29]. That study included source material from the Dicle University cohort and focused on transformer–foundation-model integration for WBC boundary delineation. The present study is not presented as an independent clinical cohort. Instead, it addresses a different technical question using overlapping source material: whether WBC segmentation can be performed effectively by combining automated cell localization with ROI-constrained diffusion and image-derived weak supervision. The relationship between the two studies, including dataset reuse and differences in methodology and experimental design, is described explicitly in Section 2. The present framework replaces the transformer–SAM2 segmentation pathway with a detection-guided diffusion strategy and evaluates its principal components through dedicated ablation experiments [29].

Accordingly, we propose a detection-guided ROI-constrained diffusion framework for weakly supervised WBC segmentation. The framework first localizes candidate WBCs using a YOLO-based detector, after which segmentation is restricted to the detector-defined ROI. Automatically generated pseudo-masks provide weak segmentation supervision during training, whereas expert masks are retained as reference annotations for validation and evaluation rather than serving as the primary segmentation-training targets. The proposed design therefore integrates cell localization, ROI restriction, pseudo-mask supervision, and diffusion-assisted segmentation within a unified processing framework.

The aim of this study was to develop and retrospectively evaluate the proposed framework using internal and independent external data. Performance was assessed separately under ROI-conditional and end-to-end evaluation, allowing segmentation capability to be distinguished from localization-dependent performance. The proposed method was additionally compared with representative CNN-, transformer-, and diffusion-based segmentation approaches, and its principal design components were examined through ablation experiments. External generalization was evaluated without dataset-specific model adaptation. These experiments are intended to characterize segmentation performance, component contributions, and robustness under the reported experimental conditions; they are not presented as prospective clinical validation or as evidence of improved diagnosis, treatment decisions, or patient outcomes.

## 2. Materials and Methods

### 2.1. Study Design and Ethical Considerations

This study was designed as a retrospective computational development and dataset-evaluation study of WBC segmentation from microscopic peripheral blood-smear images. The objective was to develop and evaluate a detection-guided region-of-interest (ROI)-constrained diffusion framework under clearly separated model-development, internal-testing, and external-testing conditions. The study was not designed as a prospective clinical trial, and no claims of diagnostic accuracy, clinical benefit, or treatment efficacy are made. Instead, the proposed framework was evaluated as a computational image-analysis method using retrospective microscopic image datasets.

**Figure 1:**
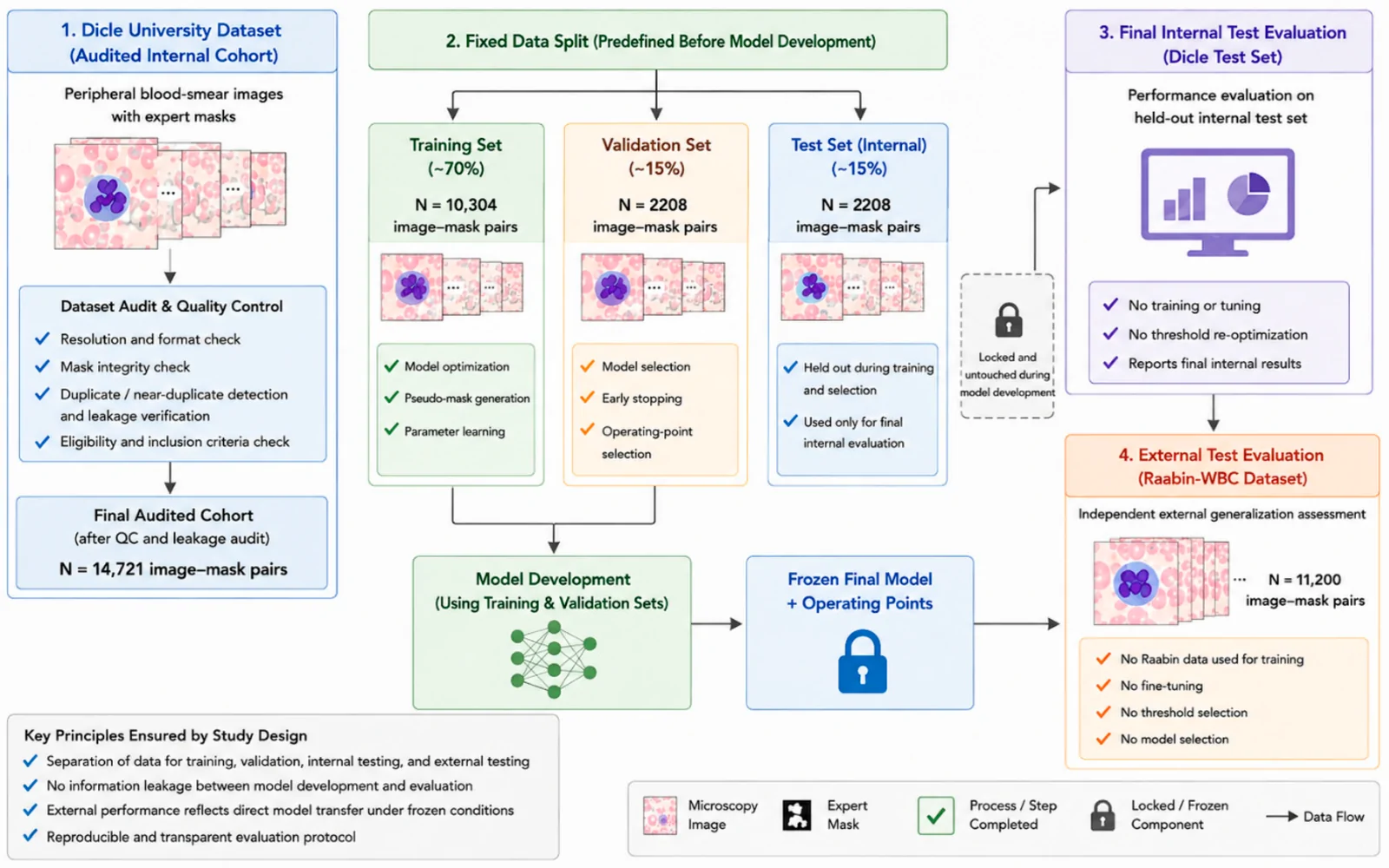
Overall study design and evaluation workflow. The Dicle University dataset underwent quality control and leakage auditing before assignment to fixed training, validation, and held-out test partitions. Training data were used for model optimization, whereas validation data were used for model selection and operating-point determination. The held-out Dicle test set was isolated during model development and used only for final internal evaluation. After the model and operating points were frozen, external evaluation was performed on 11,200 Raabin-WBC samples without retraining, fine-tuning, or Raabin-based threshold selection.

The overall study workflow is illustrated in Figure 1. Model development was performed exclusively using the internal Dicle University dataset, while external generalization was assessed independently using the Raabin-WBC dataset. To ensure a rigorous evaluation protocol, the roles of the training, validation, internal test, and external test datasets were predefined before model development. The resulting study design prevented information leakage between model development and evaluation and ensured that external performance reflected direct model transfer without additional optimization.

The internal Dicle University dataset served as the development dataset for both detector and segmentation model training. Following record-level identity verification and duplicate reconciliation, the final cohort consisted of 14,721 WBC image–mask records, equally distributed across three cell classes (Blast, Lymphocyte, and Neutrophil). The dataset was divided into a fixed 70% training (10,305 records), 15% validation (2208 records), and 15% test (2208 records) protocol. Exact duplicate image–mask groups were retained within individual partitions to prevent duplicate leakage across model-development and evaluation subsets. Because complete patient identifiers could not be reconstructed for all records, the primary evaluation is reported as a record-level in-domain evaluation rather than patient-level cross-validation.

The publicly available Raabin-WBC dataset was used exclusively for external evaluation to assess model robustness under an independent acquisition protocol and imaging distribution [3]. Throughout the study, the external dataset remained frozen and was not used for model training, fine-tuning, hyperparameter optimization, threshold selection, or model selection.

The proposed framework employed weakly supervised learning. Bounding boxes derived from expert segmentation masks were used to supervise detector training, whereas automatically generated pseudo-masks provided weak supervisory signals for segmentation. Expert pixel masks were not used as training targets for the segmentation model; instead, they were retained as reference annotations for validation and final quantitative evaluation. This distinction is important because the proposed framework is weakly supervised rather than unsupervised [8].

The present study also shares source material with our previously published TransNet–SAM2 framework [29]. However, the two studies investigate different scientific questions. The previous work evaluated a transformer–foundation-model segmentation framework, whereas the present study investigates detection-guided ROI-constrained diffusion under weak supervision. The methodological relationship between the two studies, including the extent of dataset reuse and differences in experimental design, is described in detail in Section 2.3.

A summary of the overall study design, dataset roles, supervision strategy, and evaluation protocol is provided in Table 1.

Table 1 summarizes the final experimental roles of each dataset partition. The fixed Dicle training partition contained 10,305 records and was used for detector development. Following ROI eligibility and pseudo-mask quality control, 10,129 training ROIs were retained for weakly supervised segmentation training. The 2208-record validation partition was used for model selection and operating-point determination, whereas the 2208-record test partition remained isolated until final internal evaluation. Raabin-WBC was used only for frozen external evaluation, with no external fine-tuning or parameter selection.

### 2.2. Datasets

Two datasets were used in this study. The Dicle University dataset served as the internal dataset for model development and in-domain evaluation, whereas the Raabin-WBC dataset was reserved exclusively for external evaluation. Their roles were predefined before model development and remained unchanged throughout the study, as illustrated in Figure 1. The principal characteristics and experimental roles of the two datasets are summarized in Table 2.

#### 2.2.1. Dicle University Dataset

The internal dataset was obtained from the Department of Pathology, Dicle University, Türkiye, and consists of microscopic peripheral blood-smear images collected for computational analysis of white blood cells. The source material overlaps with that used in our previously published TransNet–SAM2 study [29]. However, the present study investigates a different computational framework and uses a revised supervision and evaluation protocol. The relationship between the two studies is described in Section 2.3.

For the present study, the frozen dataset consisted of 14,721 verified WBC records. Record identities were reconciled before model development using image hashes, mask hashes, and image–mask pair signatures. Duplicate-leakage auditing identified 45 duplicate image–mask groups. These groups were kept within individual partitions, and no duplicate image–mask pairs crossed the training, validation, or test subsets.

The verified cohort contained three WBC classes: Blast, Lymphocyte, and Neutrophil, with 4907 records per class. A fixed 70%/15%/15% split was used, resulting in 10,305 training, 2208 validation, and 2208 test records. Each class contributed 3435 training, 736 validation, and 736 test records. The split was performed at the record level while preserving duplicate-group integrity. Because complete patient identifiers could not be recovered for all records, the present evaluation is reported at the record level rather than as patient-level cross-validation.

Expert segmentation masks served two distinct purposes in the revised framework. For detector training, bounding boxes were derived from the expert masks to provide reliable localization targets. For segmentation, however, expert pixel masks were not used as training targets; instead, the proposed segmentation model was trained using automatically generated pseudo-masks. Expert masks were retained as reference annotations for validation and final quantitative evaluation. The detector and segmentation supervision procedures are described in Section 2.5 andSection 2.8.

#### 2.2.2. Raabin-WBC Dataset

The Raabin-WBC dataset is a publicly available collection of peripheral blood-smear images introduced by Kouzehkanan et al. [3]. In the present study, a frozen external evaluation cohort comprising 11,200 records was used to assess cross-dataset transfer of the proposed framework under an independent image distribution.

To preserve the independence of the external evaluation, no Raabin-WBC data were used for detector training, segmentation training, fine-tuning, model selection, threshold selection, hyperparameter optimization, or early stopping. The operating points determined using the Dicle development data were frozen before external evaluation. Raabin-WBC therefore served exclusively as an external test cohort. This protocol was intended to measure direct model transfer under dataset shift without introducing information from the external cohort into model development.

The external cohort was subjected to an additional integrity audit before final analysis. All 11,200 image and mask records were available, image–mask dimensions were consistent, and reference masks were non-empty. However, reliable class metadata could not be recovered from the frozen external-evaluation records. Consequently, the revised study reports global external segmentation results only and does not present class-wise Raabin-WBC performance. This restriction avoids unsupported mapping between the Raabin-WBC categories and the Dicle study labels.

Preprocessing for the external evaluation followed the same frozen inference pipeline defined before Raabin-WBC was accessed. No dataset-specific adaptation or post hoc optimization was performed. The main characteristics and experimental roles of the internal and external cohorts are summarized in Table 2.

The audited characteristics, experimental roles, supervision sources, and data-use restrictions for the two datasets are summarized in Table 2. In particular, the Dicle University dataset was used for model development and internal evaluation, whereas Raabin-WBC remained completely independent of model optimization and was used only for frozen external evaluation.

### 2.3. Relationship to the Published TransNet–SAM2 Study

The Dicle University data used in the present study originate from the same institutional source material as our previously published TransNet–SAM2 study [29]. Accordingly, the present study is not considered an independent clinical cohort relative to that publication. The overlap concerns the underlying Dicle University WBC image collection and associated expert reference annotations. In the present study, these source data were re-audited and organized into a fixed cohort of 14,721 verified records, comprising 4907 Blast, 4907 Lymphocyte, and 4907 Neutrophil records, with fixed traın (10,305), valıdatıon (2208), and test (2208) partitions. Duplicate-group integrity was explicitly audited, and no duplicate image–mask pairs crossed these partitions.

Despite the shared source material, the two studies address different methodological questions and use different computational pipelines. The published TransNet–SAM2 study investigated WBC segmentation through transformer-based feature extraction and SAM2-based boundary delineation. In contrast, the present study evaluates a YOLOv13-N-guided, ROI-constrained diffusion-assisted segmentation framework under weak supervision. Here, detector localization precedes segmentation, and the segmentation model is trained using automatically generated pseudo-masks rather than expert pixel masks as its primary training targets. Expert masks are retained for validation and final evaluation. The present study additionally introduces a fixed held-out evaluation protocol, pseudo-mask quality assessment, component-level ablation, ROI-conditional and end-to-end evaluation, and frozen external validation on 11,200 Raabin-WBC records without external model adaptation.

Thus, reuse of the Dicle source material should not be interpreted as an independent replication cohort. The scientific contribution evaluated here is the detection-guided, ROI-constrained weak-supervision strategy and its internal and external performance characteristics, rather than the introduction of a new clinical dataset.

### 2.4. Overall Framework

**Figure 2:**
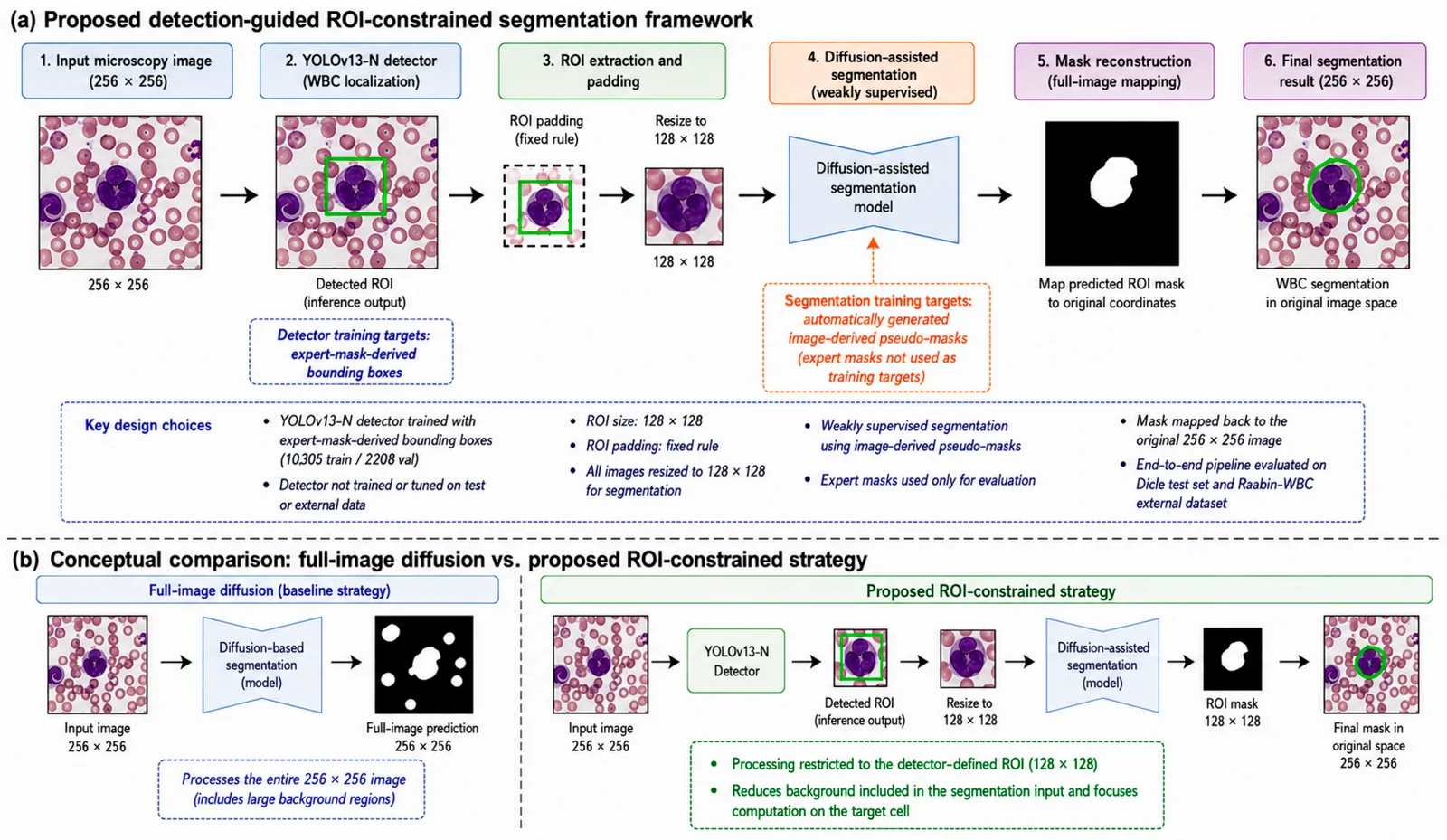
Overview of the proposed detection-guided ROI-constrained segmentation framework. (**a**) Processing pipeline showing YOLOv13-N localization, ROI extraction and resizing, diffusion-assisted weakly supervised segmentation, and reconstruction of the predicted mask in the original image space. Expert-mask-derived bounding boxes were used as detector training targets, whereas image-derived pseudo-masks were used to train the segmentation component. (**b**) Conceptual comparison between full-image diffusion and the proposed ROI-constrained strategy. The proposed approach restricts segmentation to the detector-defined ROI; its contribution to segmentation performance is evaluated separately in the ablation study.

The proposed framework combines WBC localization with ROI-constrained weakly supervised segmentation. As illustrated in Figure 2a, each 256 × 256 microscopy image is first processed by a YOLOv13-N detector to localize the target white blood cell. In the revised experimental pipeline, the detector was trained using bounding boxes derived from expert segmentation masks in the Dicle training partition. The expert masks were used only to obtain reliable localization targets for the detector and were not used as pixel-level training targets for the segmentation model. The final detector supervision therefore differs from the earlier pseudo-box formulation evaluated during method development.

For each detected cell, the bounding box is expanded using the predefined ROI-padding rule, and the resulting region is resized to 128 × 128 pixels. The ROI is then processed by the diffusion-assisted segmentation model. Segmentation training uses automatically generated image-derived pseudo-masks rather than expert pixel masks, preserving the weakly supervised nature of the segmentation stage. The predicted ROI mask is subsequently mapped back to its original coordinates to obtain the final full-image segmentation result. The 128 × 128 ROI processing and reconstruction of the mask at its original image location follow the core ROI-constrained design of the framework.

Figure 2a summarizes the complete processing sequence: input microscopy image → YOLOv13-N localization → ROI extraction → diffusion-assisted segmentation → mask reconstruction. The separation between localization and segmentation is important because the segmentation model operates only within detector-derived ROIs. Consequently, localization errors or missed detections can directly affect the final end-to-end segmentation result. This dependency is evaluated explicitly in the held-out Dicle test analysis and the Raabin-WBC external failure analysis.

Figure 2b illustrates the conceptual difference between conventional full-image diffusion and the proposed ROI-constrained strategy. Instead of processing the entire microscopy patch, the proposed method restricts diffusion-based segmentation to the detector-localized region. This design reduces unnecessary background processing and focuses the segmentation stage on the target cell. The contribution of ROI restriction and the diffusion component is evaluated separately through the ablation experiments rather than assumed from the architecture alone.

### 2.5. YOLOv13-N Detection and ROI Extraction

WBC localization was performed using the Nano configuration of YOLOv13 (YOLOv13-N) [30], implemented using the official YOLOv13 codebase. The detector was initialized from the pretrained yolov13n.pt checkpoint and configured as a single-class WBC detector. Detector inputs were resized to 256 × 256 pixels. The instantiated training model contained approximately 2.46 million parameters and required approximately 6.4 GFLOPs. Model development was performed using Python 3.12.13, PyTorch 2.11.0 with CUDA 12.8, and the YOLOv13 implementation based on Ultralytics 8.3.63.

The detector was trained for a maximum of 100 epochs using AdamW [31] with an initial learning rate of 1 × 10^−4^, weight decay of 5 × 10^−4^, momentum of 0.937, and a batch size of 16. A cosine learning-rate schedule was applied with 10 warm-up epochs, and early-stopping patience was set to 20 epochs. Training used a fixed random seed of 42 with deterministic execution enabled. Automatic mixed-precision training was used. The detector loss comprised bounding-box regression, classification, and distribution focal loss terms, with corresponding loss weights of 7.5, 0.5, and 1.5, respectively.

Training-time augmentation included HSV perturbation (hsv_h = 0.015, hsv_s = 0.7, hsv_v = 0.4), horizontal flipping with probability 0.5, translation of 0.1, scaling of 0.5, Mosaic augmentation with probability 1.0, and copy–paste augmentation with probability 0.1; MixUp was disabled. Mosaic augmentation was disabled during the final 10 epochs. The training pipeline additionally applied low-probability blur, median blur, grayscale conversion, and CLAHE transformations, together with JPEG compression augmentation.

The detector was trained using the fixed dicle traın partition, and model selection was performed using the dicle valıdatıon partition; the held-out dicle test set and Raabin-wbc external cohort were not used for detector optimization. The best validation checkpoint was retained for downstream roı generation. For each detected wbc, the predicted bounding box was expanded using the predefined 25% roı-padding rule, and the resulting region was cropped from the original microscopy image and resized to 128 × 128 pixels before segmentation. The same frozen detector and ROI-generation procedure was subsequently applied during internal held-out and external evaluation without dataset-specific detector retraining or adaptation.

### 2.6. Automatic Pseudo-Mask Generation

The segmentation model was trained using automatically generated pseudo-masks rather than expert pixel-level masks. For each Dicle training image $i_{i}$, the YOLOv13-N detector first localized the target WBC. The detected bounding box was expanded using the fixed ROI-padding rule, and the resulting region was resized to 128 × 128 pixels to obtain the ROI, $R_{i}$.

The final pseudo-mask procedure followed the frozen dicle_image_only_pseudomask configuration. All candidate masks were generated directly from the 128 × 128 detector-derived ROI image, without access to the corresponding expert segmentation mask. Four image-derived candidate procedures were considered: the baseline image-thresholding procedure, border-color-distance segmentation, saturation–darkness segmentation, and a GrabCut center-prior procedure. A fifth candidate, termed auto_ensemble, combined these image-derived masks using a predefined majority-consensus rule with image-only component scoring. The resulting binary ensemble mask was used as the final weak segmentation target.

The ensemble policy was established using a predefined 600-record training-only diagnostic cohort, balanced at 200 records per WBC class, and was subsequently frozen before generation of the complete training pseudo-mask set. Expert masks were not used for candidate-mask generation, component scoring, method selection, or final automatic mask selection; they were used only for post hoc quality assessment of the training-only diagnostic cohort. Neither the validation set, held-out Dicle test set, nor Raabin-WBC data contributed to selection of the pseudo-mask policy. After freezing the procedure, it was applied to all 10,129 eligible Dicle training ROIs, producing a non-empty pseudo-mask for every eligible ROI.

Pseudo-masks were generated automatically from the image content within each ROI. Because WBC appearance varies across cell types and staining conditions, multiple image-derived candidate masks were considered and combined using the fixed automatic ensemble/rescue procedure established on the training data. The resulting binary pseudo-mask can be expressed as:

$$C\Rightarrow P\left(R_{i}\right)$$

where P(.) denotes the frozen image-based pseudo-mask generation procedure and ${\bar{M}}_{i}\in {\left[0,1\right]}^{128\;by\;128}$ is the weak segmentation target.

Importantly, detector and segmentation supervision were kept distinct. Expert masks were used to derive bounding-box targets for detector training, whereas the segmentation network was optimized using the automatically generated pseudo-masks:

$$\left(R_{i},{\bar{M}}_{i}\right)\to segmentation\;training$$

Expert pixel masks were not used as segmentation-training targets and were retained for validation and final evaluation. No validation, held-out Dicle test, or Raabin-WBC data were used to generate the training pseudo-masks or optimize the pseudo-mask generation policy.

### 2.7. ROI-Constrained Diffusion Framework

The segmentation stage applies diffusion-based learning only within the detector-defined WBC ROI rather than to the complete microscopy image. Each padded ROI is resized to 128 × 128 pixels and paired with the automatically generated pseudo-mask described in Section 2.5. This restricts segmentation learning to the localized cellular region while reducing the amount of surrounding background presented to the model.

The diffusion backbone follows a U-Net encoder–decoder architecture with four resolution levels and channel dimensions of 64, 128, 256, and 512. Each level contains residual blocks with group normalization and Swish activation, while skip connections transfer spatial information between corresponding encoder and decoder levels. Self-attention is incorporated at the deeper feature levels, and sinusoidal timestep embeddings condition the network on the diffusion step. These architectural elements follow the configuration used in the original framework.

During forward diffusion, Gaussian noise is progressively added to the ROI representation. For an ROI $x_{0}$, the noisy representation at timestep t is:

$$x_{t}=\sqrt{\alpha_{t}x_{0}}+\sqrt{1-\alpha_{t}}\epsilon ,\epsilon ∼N\left(0,1\right),$$

where $\Pi_{s=1}^{t}\left(1-\beta_{s}\right)\;\mathrm{and}\;\beta_{t}$ defines the noise Schedule. The denoising network $\in_{\theta }(x_{t},t)$ is trained to estimate the noise introduced at each timestep:

$$L_{diff}=E_{x_{0},t,\epsilon }[∥\epsilon -\epsilon_{\theta }\left(x_{t},t\right)∥]2^{2}$$

In parallel, the segmentation branch predicts the WBC mask within the ROI and is optimized using the image-derived pseudo-mask $\bar{M}$ as the weak pixel-level target. Expert segmentation masks are not used as optimization targets for this branch. The diffusion and segmentation objectives are combined during joint training, allowing the network to learn denoising representations while simultaneously learning the ROI-level segmentation task.

At inference, the trained model produces a probability map for each detector-defined ROI. The ROI prediction is converted to a binary mask using the operating point selected on the Dicle validation partition and is subsequently mapped back to the corresponding coordinates of the original 256 × 256 microscopy image. No test or Raabin-WBC data are used to select the segmentation operating point.

### 2.8. Loss Functions

The proposed model was optimized using complementary segmentation and diffusion objectives. Because segmentation was weakly supervised, the automatically generated pseudo-mask M~ described in Section 2.5 was used as the training target rather than the expert reference mask.

The segmentation loss combines binary cross-entropy (BCE) and Dice loss:

$$L_{seg}=0.25L_{BCE}+0.75L_{Dice}$$

where

$$L_{BCE}=-\frac{1}{N}\sum_{i=1}^{N}[{\bar{M}}_{i}\log\left(p_{i}\right)+\left(1-{\bar{M}}_{i}\right)log(1-p_{i})],$$

and

$$L_{Dice}=1-\frac{2\sum_{i}p_{i}{\bar{M}}_{i}+\epsilon }{\sum_{i}p_{i}+\sum_{i}{\bar{M}}_{i}+\epsilon }$$

Here, $P_{i}$ denotes the predicted foreground probability, $\bar{M}$ is the corresponding pseudo-mask label, and *ϵ* is a small constant for numerical stability. BCE provides pixel-wise supervision, whereas Dice loss accounts for overlap between the predicted and pseudo-mask regions.

The diffusion branch was optimized by predicting the Gaussian noise added at timestep t:

$$L_{diff}=E_{x_{0},t,\epsilon }[∥\epsilon -\epsilon_{\theta }\left(x_{t},t\right)∥]2^{2}.$$

During joint optimization, the two objectives were combined as:

$$L_{total}=0.25L_{seg}+0.20L_{diff}$$

Thus, segmentation remained the primary optimization objective, while the diffusion term provided an auxiliary denoising objective. The final training configuration used image-derived pseudo-masks for segmentation supervision and expert masks only as validation references; the held-out test data were not used for optimization.

### 2.9. Training Strategy

The proposed segmentation model was trained using a two-stage strategy. In the first stage, the segmentation branch was optimized for 10 warm-up epochs using the image-derived pseudo-masks described in Section 2.5. This initialization allowed the network to learn a stable ROI-level segmentation representation before introducing the diffusion objective. In the second stage, segmentation and conditional diffusion were optimized jointly for up to 120 epochs, with early stopping based only on performance on the Dicle validation subset. The final training run used 10,129 eligible training ROIs and 2164 validation ROIs, while the held-out Dicle test set and Raabin-WBC dataset were not accessed during training or model selection.

All ROI inputs were resized to 128 × 128 pixels. During training, the segmentation branch received the ROI image together with its automatically generated pseudo-mask, whereas expert masks were used only as validation references. The segmentation loss and diffusion loss defined in Section 2.7 were optimized jointly during the second phase. Model checkpoints were evaluated on the validation subset, and the checkpoint with the highest validation Dice score was retained. The joint phase used an early-stopping patience of 20 epochs without improvement, preventing unnecessary continuation once validation performance ceased to improve.

After training, the segmentation operating point was selected using the validation subset only and then frozen before held-out testing. No threshold, test-time augmentation policy, post-processing rule, or model checkpoint was selected using the Dicle test set or Raabin-WBC data. The final frozen model therefore remained independent of both internal and external test evaluation.

### 2.10. Evaluation Protocol

The proposed framework was evaluated using predefined internal and external protocols. Model development was restricted to the Dicle training and validation partitions. The held-out Dicle test set was accessed only after the model and operating points had been frozen and was not used for training, model selection, threshold selection, or fine-tuning.

Segmentation performance was evaluated against expert reference masks using the Dice similarity coefficient (Dice), intersection over union (IoU), precision, and recall. Because segmentation depends on detector-generated ROIs, two evaluation levels were considered. ROI-conditional evaluation assessed segmentation when a valid target ROI was available, whereas end-to-end evaluation assessed the complete localization-to-segmentation pipeline and therefore included the effect of missed or inadequate detections.

Ablation experiments were performed under the same frozen evaluation conditions to assess the contribution of the principal framework components. Comparisons between segmentation-only and diffusion-assisted configurations used paired sample-level predictions, without test-set optimization.

External generalization was evaluated on 11,200 Raabin-WBC samples using the model and operating points frozen from Dicle development. No Raabin data were used for training, fine-tuning, model selection, threshold calibration, or post-processing selection. Because reliable class metadata could not be recovered for the frozen external cohort, Raabin-WBC performance was reported at the global cohort level rather than by cell subtype.

For paired model comparisons, performance differences were summarized using 95% bootstrap confidence intervals, with the Wilcoxon signed-rank test used for non-parametric paired statistical testing.

## 3. Results

### 3.1. Dataset Audit and Experimental Cohort

The final dataset audit identified 14,721 verified Dicle WBC records, equally distributed among Blast, Lymphocyte, and Neutrophil (4907 records per class). The fixed dataset split comprised 10,305 training, 2208 validation, and 2208 held-out test records. Each class contributed 3435 training, 736 validation, and 736 test records.

Identity and duplicate auditing identified 45 duplicate image–mask groups. These groups were contained within individual partitions, and the final audit found no duplicate image–mask or sample-identity leakage across the training, validation, and test partitions. Following ROI eligibility and pseudo-mask quality control, 10,129 training ROIs were retained for weakly supervised segmentation training.

The external evaluation cohort comprised 11,200 Raabin-WBC records. The Dicle test partition remained excluded from model development until final internal evaluation, while Raabin-WBC was used only for frozen external evaluation. Neither cohort contributed to model training, fine-tuning, or model selection, and no Raabin-based operating-point optimization was performed.

### 3.2. Detector Localization Performance

The final YOLOv13-N detector was evaluated on the 2208-record Dicle validation partition. Unlike the detector reported in the original manuscript, the revised detector was trained using expert-mask-derived bounding boxes rather than automatically generated pseudo-boxes. The held-out Dicle test set was not accessed for detector training, model selection, or threshold selection.

The frozen detector achieved a precision of 0.9151, recall of 0.8949, and F1-score of 0.9049. The corresponding mAP50 was 0.9516, while mAP50–95 was 0.7423 (Table 3). These results indicate that the revised detector provided reliable localization on the internal development data and supported its use for ROI extraction in the subsequent segmentation pipeline.

Overall, the final detector showed high localization performance, with both precision and recall approaching 0.90 and an mAP50 of 0.9516. These results support its use for generating the ROIs required by the subsequent segmentation stage.

### 3.3. Quality of the Automatically Generated Training Pseudo-Masks

The automatic pseudo-mask pipeline generated valid non-empty masks for all 10,129 eligible Dicle training ROIs (100%). To assess the quality of these weak supervisory labels, 600 training records were independently compared with the corresponding expert reference masks. Of these, 590 contained expert-positive reference regions.

Across the verification cohort, the generated pseudo-masks achieved a mean Dice of 0.7020, median Dice of 0.7234, and mean IoU of 0.5717. Mean precision was 0.8578, whereas mean recall was 0.6766. The expert-positive detection rate was 1.000, and the severe-mismatch rate was 3.22%. These findings show (Table 4) that the automatic procedure generally identified the target cellular regions but did not reproduce expert boundaries perfectly, supporting their intended role as weak training labels rather than substitutes for expert annotations.

Pseudo-mask quality also varied among WBC classes. Lymphocytes showed the highest agreement with expert annotations, followed by Blasts and Neutrophils. The lower recall observed particularly for Neutrophils indicates that the automatic masks tended to capture only part of the expert-defined cell region in more difficult cases.

The class-wise analysis confirms that pseudo-mask quality was morphology-dependent, with the strongest agreement for Lymphocytes and the lowest for Neutrophils. These results further support treating the generated masks as imperfect weak supervision rather than expert-equivalent annotations.

### 3.4. Held-Out Dicle Segmentation Performance

The final proposed model was evaluated on the 2208-record held-out Dicle test set after model selection and operating points had been frozen. Of these records, 2164 had a valid detector-generated ROI, corresponding to an internal detector ROI coverage of 98.01%. No training, fine-tuning, threshold selection, post-processing selection, or test-time augmentation selection was performed using the test data.

Two complementary evaluations were performed. Under ROI-conditional evaluation, which measures segmentation performance when a valid detector ROI was available, the proposed model achieved a mean Dice of 0.7188, IoU of 0.5796, precision of 0.8966, and recall of 0.6422. Under the stricter end-to-end evaluation, which includes detector failures, Dice decreased to 0.6705, with an IoU of 0.5408, precision of 0.8378, and recall of 0.5986. These results are summarized in Table 5. 

The difference between the two evaluation levels corresponded to a Dice penalty of 0.0483 when detector misses were incorporated into the complete pipeline. This finding demonstrates the importance of reporting end-to-end performance in addition to segmentation performance conditional on successful localization.

### 3.5. Ablation Study

An ablation study was conducted on the held-out Dicle test set to quantify the contribution of the diffusion component to segmentation performance. The segmentation-only configuration and the full diffusion-assisted configuration were evaluated under the same frozen evaluation protocol. No model retraining, threshold optimization, test-time augmentation selection, or model selection was performed using the test data.

As shown in Table 6, the segmentation-only configuration achieved a mean Dice score of 0.7263, whereas the full diffusion-assisted configuration achieved 0.7188. Across the 2063 paired expert-positive test samples, the mean paired Dice difference was −0.0075, with a 95% bootstrap confidence interval of −0.0083 to −0.0066. The corresponding two-sided Wilcoxon signed-rank test yielded W = 533,839 and *p* = 3.61 × 10^−81^.

**Figure 3:**
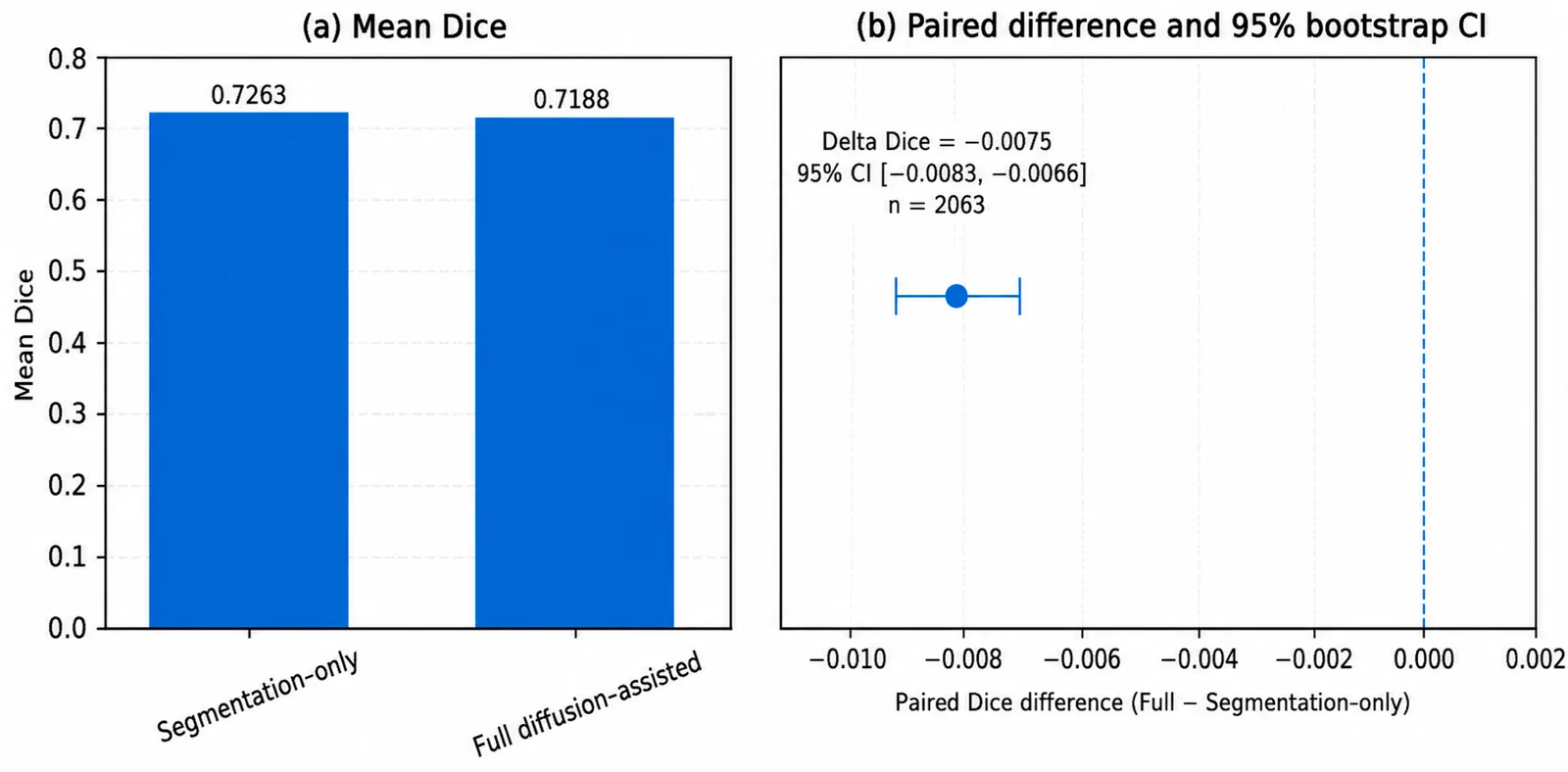
Ablation analysis of the diffusion component on the held-out Dicle test set. (**a**) Mean Dice scores of the segmentation-only and full diffusion-assisted configurations. (**b**) Mean paired Dice difference (full diffusion-assisted–segmentation-only) across 2063 paired expert-positive samples, with a 95% bootstrap confidence interval. The full model produced a mean paired difference of −0.0075 (95% CI: −0.0083 to −0.0066).

The paired comparison is further illustrated in Figure 3. The distribution of Dice differences was shifted slightly below zero, indicating that incorporation of the diffusion component did not provide an incremental improvement in mean Dice under the evaluated configuration. The magnitude of the difference was small, with an absolute reduction of approximately 0.75 percentage points. Thus, the ablation results indicate that the segmentation backbone accounts for the principal segmentation performance observed in this experiment, while the evaluated diffusion component did not yield additional Dice improvement.

### 3.6. Statistical Comparison on the Held-Out Dicle Test Set

Paired analysis was performed on the 2063 expert-positive records of the held-out Dicle test set for which both ablation configurations were evaluated. The full diffusion-assisted configuration achieved a mean Dice of 0.7188 compared with 0.7263 for the segmentation-only configuration, corresponding to a mean paired Dice difference of −0.0075 (95% bootstrap CI: −0.0083 to −0.0066). A two-sided Wilcoxon signed-rank test confirmed that the paired difference was statistically significant (W = 533,839; *p* = 3.61 × 10^−81^). However, the magnitude of the difference was small, corresponding to less than one percentage point in Dice.

The statistical unit in this analysis was the individual image record. Because complete patient identifiers were not available for the frozen cohort, patient-level clustering or resampling could not be performed. The reported interval therefore characterizes uncertainty at the record level and should not be interpreted as patient-level inference.

### 3.7. External Validation on Raabin-WBC

External validation was performed on the frozen Raabin-WBC cohort of 11,200 records using the Dicle-trained model and operating points without external adaptation. No Raabin data were used for training, fine-tuning, model selection, threshold selection, detector calibration, test-time augmentation selection, or post-processing selection.

The detector generated a valid ROI for 2071 of 11,200 records, corresponding to an ROI coverage of 18.49%. Among successfully localized cases, the full diffusion-assisted model achieved an ROI-conditional Dice of 0.3760. When detector failures were incorporated into the complete evaluation pipeline, the end-to-end Dice was 0.0411, with an IoU of 0.0253, precision of 0.1538, and recall of 0.0255.

The marked difference between ROI-conditional and end-to-end performance indicates that localization failure was a major limitation of external transfer. The external data-integrity audit confirmed complete image and mask availability, matching image–mask dimensions, and non-empty reference masks; therefore, the low end-to-end performance primarily reflects limited transfer of the Dicle-trained pipeline to the independent Raabin-WBC domain rather than missing evaluation data.

Class-wise external performance was not reported because reliable cell-class metadata could not be reconstructed for the frozen Raabin-WBC evaluation cohort. Consequently, external evaluation was restricted to cohort-level metrics to avoid potentially incorrect retrospective class assignments.

### 3.8. External Failure Analysis

A dedicated failure analysis was performed to determine the main source of performance degradation during frozen Raabin-WBC evaluation. The audit showed that the principal limitation occurred at the localization stage. The Dicle-trained detector generated valid ROIs for only 2071 of 11,200 external records (18.49%), while 9129 records (81.51%) had no usable detector ROI.

The effect of localization failure was reflected in the segmentation results. When evaluation was restricted to successfully localized cases, the full diffusion-assisted model achieved an ROI-conditional Dice of 0.3760. When all external records, including detector failures, were included, the end-to-end Dice decreased to 0.0411. This large difference indicates that failure to transfer the detector-defined ROI mechanism was a major bottleneck in the external pipeline.

Among the full-model predictions, the frozen failure inventory identified 1349 severe segmentation failures, while 570, 136, and 16 records were categorized as low-, moderate-, and high-agreement cases, respectively. The much larger group of 9129 detector ROI failures therefore preceded segmentation and constituted the dominant external failure category.

**Figure 4:**
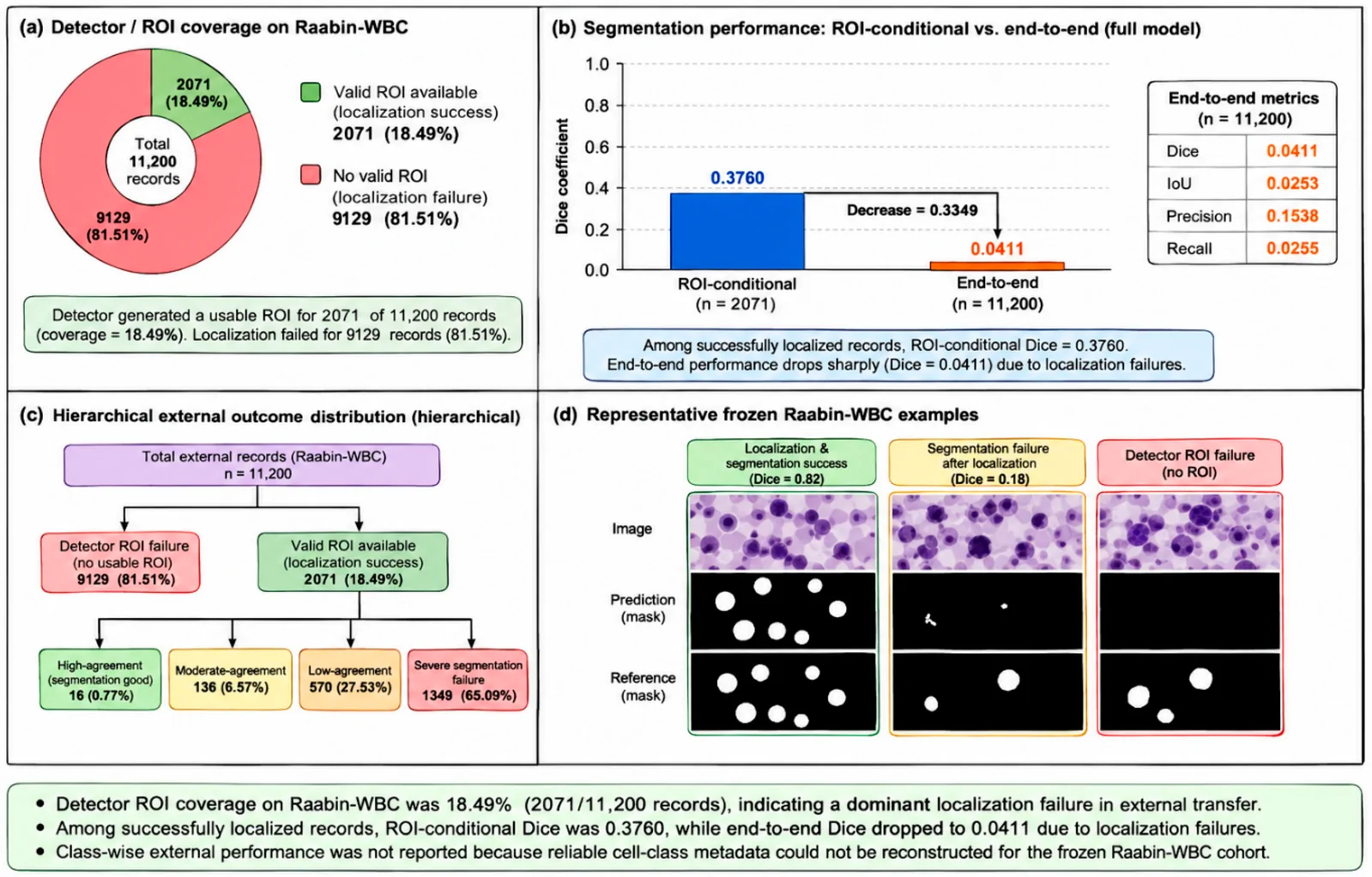
External validation and failure analysis on the frozen Raabin-WBC cohort. (**a**) Detector ROI availability, showing successful localization in 2071 of 11,200 records (18.49%) and localization failure in 9129 records (81.51%). (**b**) Comparison of ROI-conditional Dice (0.3760) and end-to-end Dice (0.0411) for the full diffusion-assisted model. (**c**) Distribution of external failure categories, demonstrating that detector ROI failure was the dominant failure mechanism. (**d**) Representative external cases illustrating successful localization and segmentation, segmentation failure after localization, and detector localization failure. Representative examples should be selected using a predefined rule rather than subjective visual selection.

Basic data-integrity problems were ruled out as an explanation for this degradation. All 11,200 external records had available images and reference masks, 100% image–mask dimension agreement, and 100% non-empty reference masks. The audit therefore identified the external behavior as consistent with domain shift or target-representation mismatch, rather than missing or structurally invalid evaluation data (Figure 4).

### 3.9. Qualitative Segmentation Analysis

Qualitative analysis was performed to complement the quantitative evaluation and to illustrate representative segmentation behavior under both internal and external conditions. Examples were selected using predefined performance-based criteria rather than manual visual selection. For the Dicle test set, cases were sampled to represent successful and difficult segmentations according to their sample-level Dice scores. For Raabin-WBC, examples were selected separately from successfully localized cases and from the predefined external failure categories described in Section 3.8.

**Figure 5:**
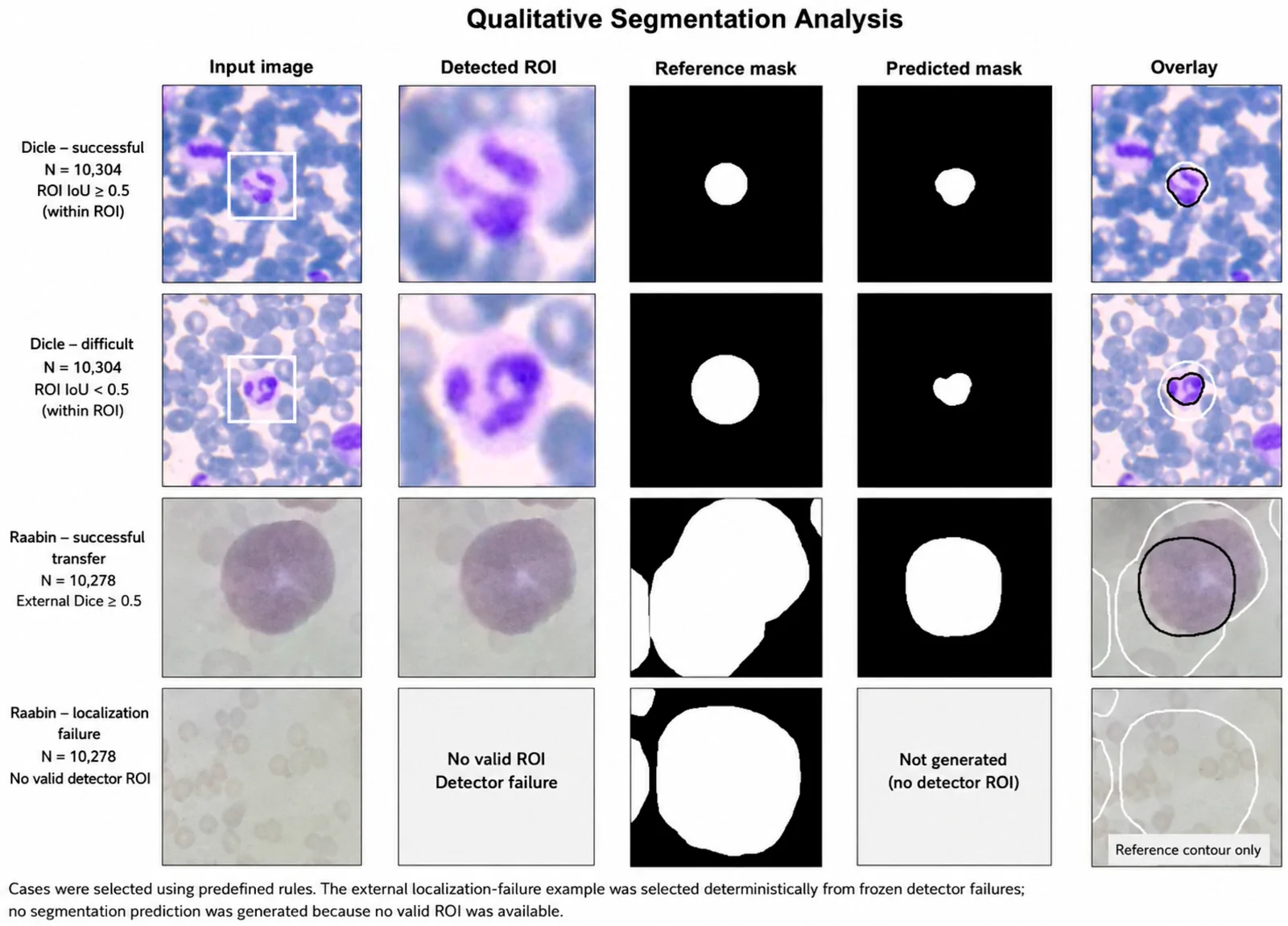
Qualitative segmentation analysis on the held-out Dicle test set and external Raabin-WBC dataset. Representative cases were selected using prespecified performance-based criteria rather than subjective visual selection. Each example shows the input image, detected ROI, expert reference mask, predicted mask, and corresponding overlay. Dicle examples represent successful and difficult internal cases, whereas Raabin-WBC examples illustrate successful external transfer and representative localization/segmentation failure patterns.

Figure 5 presents each case using the same sequence: input image → detected ROI → expert/reference mask → predicted mask → prediction-reference overlay. On the held-out Dicle set, successful cases generally showed close agreement between predicted and reference cell regions, whereas difficult cases exhibited incomplete boundaries, under-segmentation, or localization-related errors. In the external Raabin-WBC cohort, some successfully localized cells remained segmentable despite the acquisition-domain shift; however, localization failures and poor segmentation following localization were also observed, consistent with the quantitative external failure analysis.

### 3.10. Computational Characteristics

The final diffusion-assisted segmentation model contained 11,018,362 trainable parameters and operated on detector-derived ROIs resized to 128 × 128 pixels. Final held-out testing was performed on an NVIDIA A100-SXM4-80GB GPU using the frozen segmentation threshold, four-way-flip test-time augmentation, and no post-processing.

For the complete locked Dicle test evaluation of 2208 records, the recorded runtime was 38.58 min. This measurement reflects execution of the frozen evaluation pipeline under the experimental implementation and should therefore not be interpreted as a hardware-independent per-image latency benchmark.

Peak GPU memory consumption, FLOPs, and independently benchmarked per-patch inference latency were not retained as validated measurements in the frozen experimental package and are therefore not reported. In particular, the previously reported 48 ms/image, 62 million parameters, and 9.1 GB GPU-memory values should not be carried into the revised Results because those values originate from the earlier manuscript rather than the frozen V3 evaluation. The revised analysis also makes no extrapolation from patch-level computation to whole-smear or clinical-workflow processing time.

### 3.11. Comparison with Baseline Segmentation Models

The proposed framework was compared with five reference segmentation models on the fixed 2208-record Dicle test cohort. Table 7 summarizes the recovered frozen results. TransUNet achieved the highest reference Dice (0.8566), followed by Swin-Unet (0.8155), Attention U-Net (0.8053), and U-Net (0.8047), whereas the MedSegDiff-style baseline achieved 0.6531.

The proposed framework achieved a Dice of 0.7188 under ROI-conditional evaluation and 0.6705 under end-to-end evaluation. Its ROI-conditional precision (0.8966) was the highest among the evaluated configurations, indicating high reliability of positive pixel predictions when localization was successful. In contrast, the lower recall (0.6422) reduced the overall Dice score. The decrease from ROI-conditional to end-to-end performance further demonstrates the influence of detector localization on the complete pipeline.

Overall, the comparison shows that the proposed framework does not maximize internal Dice relative to the strongest reference architectures. Rather, its distinguishing characteristic is the integration of detector-guided ROI processing with pseudo-mask-based weak segmentation supervision, for which expert pixel masks are retained for evaluation rather than used as the primary segmentation-training targets.

## 4. Discussion

### 4.1. Principal Findings

This study developed and evaluated a detector-guided, weakly supervised framework for WBC segmentation that combines YOLOv13-N localization with ROI-constrained diffusion-assisted segmentation. Automatically generated pseudo-masks were used as segmentation-training targets, reducing reliance on expert pixel-level annotations during model development. On the held-out Dicle test set, the framework achieved a Dice score of 0.7188 under ROI-conditional evaluation and 0.6705 under end-to-end evaluation, highlighting the distinction between segmentation performance when a valid ROI is available and the performance of the complete detection–segmentation pipeline.

Frozen external evaluation on Raabin-WBC further demonstrated that generalization was affected by cross-dataset variation, with a greater performance reduction at the end-to-end level than when segmentation was evaluated conditionally on valid ROIs. Together with the external failure analysis, this finding identifies localization and ROI transfer as important determinants of overall performance under dataset shift, rather than attributing external degradation solely to the segmentation stage.

### 4.2. Weak Supervision and ROI-Constrained Segmentation

The pseudo-mask analysis showed that automatically generated masks provided useful, although imperfect, supervision, with a mean Dice of approximately 0.702 against expert reference masks. This level of agreement supports their use as weak training targets while also indicating the presence of annotation noise that may constrain the achievable segmentation accuracy. Importantly, expert masks were retained for reference and evaluation rather than serving as the primary segmentation-training targets, preserving the weakly supervised character of the framework.

The ROI-constrained evaluation further clarified the behavior of the proposed approach. When segmentation was assessed only for successfully localized ROIs, the model achieved a Dice of 0.7188 and a precision of 0.8966, indicating reliable foreground identification within valid detector-defined regions. End-to-end Dice decreased to 0.6705 when localization was incorporated into the complete pipeline. This difference demonstrates that overall segmentation performance depends not only on mask prediction within the ROI but also on the detector’s ability to localize the target cell correctly, making localization quality an important determinant of end-to-end performance.

### 4.3. Comparison with Baseline Models and Ablation Findings

Comparison with the reference segmentation architectures showed that TransUNet achieved the highest internal Dice (0.8566), while U-Net, Attention U-Net, and Swin-Unet also obtained higher Dice scores than the proposed framework. In contrast, the proposed model achieved the highest reported precision under ROI-conditional evaluation (0.8966), with a Dice of 0.7188. These findings indicate that the principal contribution of the framework is not superior internal segmentation accuracy alone, but the integration of detector-guided ROI processing with pseudo-mask-based weak supervision.

The ablation analysis further clarified the contribution of the diffusion component. The paired comparison between the segmentation-only configuration and the full diffusion-assisted framework did not demonstrate a substantial improvement in overall Dice attributable to diffusion. Rather, the results indicate that its contribution should be interpreted within the complete ROI-constrained framework rather than as an independent source of segmentation gain. Accordingly, the present findings support the proposed framework as a weakly supervised detection–segmentation strategy, while avoiding claims of performance improvement for individual components that were not substantiated by the controlled ablation.

### 4.4. External Generalization and Failure Analysis

External generalization was assessed on all 11,200 Raabin-WBC records using the frozen framework without dataset-specific retraining, fine-tuning, or threshold adaptation. Performance decreased relative to the held-out Dicle test set, demonstrating the challenge of transferring the framework across microscopy datasets with different acquisition and image characteristics. Importantly, the external evaluation distinguished ROI-conditional performance from end-to-end performance, allowing the effects of segmentation and localization to be considered separately.

The larger degradation at the end-to-end level indicates that detector/ROI transfer was an important bottleneck under dataset shift. When valid ROIs were available, the segmentation stage retained greater performance than the complete pipeline, whereas missed or inaccurate localization propagated directly to the final segmentation output. Thus, the external results suggest that improving cross-dataset localization robustness is particularly important for strengthening the generalizability of the overall framework.

The accompanying integrity analysis did not identify basic image–mask pairing or file-integrity problems that could account for the observed degradation, supporting dataset shift and localization sensitivity as more plausible explanations. This interpretation was also consistent with the prespecified qualitative examples, which showed successful transfer when localization was adequate and substantial segmentation degradation in difficult cases associated with inaccurate ROI localization. Together, the quantitative and qualitative analyses provide a more informative characterization of external failure than aggregate performance metrics alone.

### 4.5. Model Complexity and Inference Efficiency

The proposed segmentation model contained approximately 10.95 million parameters and operated on detector-derived ROIs resized to 128 × 128 pixels. Computational performance was assessed using the frozen inference configuration on the specified GPU device, with inference time reported from direct measurements rather than extrapolated to whole-smear processing. These characteristics indicate a relatively compact ROI-based processing strategy, while conclusions regarding clinical throughput or whole-slide efficiency are not drawn from patch-level measurements.

### 4.6. Limitations and Future Directions

Several limitations should be considered when interpreting the findings. First, the end-to-end framework remains dependent on accurate detector localization, and localization errors become more influential under cross-dataset shift. Second, the automatically generated pseudo-masks provide imperfect supervision, which may limit segmentation performance despite reducing dependence on expert pixel-level annotations. The internal dataset was restricted to three WBC classes, while the absence of reliable subtype metadata in Raabin-WBC prevented class-wise external analysis. In addition, statistical inference was conducted at the record level where patient-level identifiers were unavailable, and external validation was limited to a single independent dataset. Finally, the present evaluation was conducted on the audited WBC record cohorts and did not separately evaluate detector false-positive behavior in WBC-negative microscopic fields. Accordingly, the reported results characterize the evaluated WBC segmentation setting and should not be extrapolated to unrestricted whole-smear screening.

Future work should therefore focus on improving cross-domain localization and pseudo-label quality while extending evaluation to more diverse cell types and imaging conditions. Multicenter datasets with reliable patient- and cell-level metadata would enable stronger patient-level analysis and assessment of subtype-specific generalization. Prospective validation will also be necessary to establish the robustness of the framework across acquisition systems and clinically representative settings.

### 4.7. Overall Interpretation

The proposed framework demonstrates the feasibility of combining detector-guided ROI processing with pseudo-mask-based weak supervision for WBC segmentation. Although the approach did not exceed the strongest reference architectures in internal Dice, it achieved high ROI-conditional precision and enabled explicit evaluation of localization-dependent and end-to-end performance. The external analysis further identified localization transfer as an important limitation under dataset shift, providing a clear direction for improving the robustness of the framework.

## 5. Conclusions

This study presented a detector-guided, ROI-constrained framework for weakly supervised white blood cell segmentation. By combining YOLOv13-N localization with automatically generated pseudo-mask supervision and diffusion-assisted segmentation, the framework reduces reliance on expert pixel-level annotations while explicitly separating localization from segmentation. On the held-out Dicle test set, the proposed method achieved a Dice score of 0.7188 under ROI-conditional evaluation and 0.6705 end-to-end, with a high ROI-conditional precision of 0.8966. The pseudo-mask analysis further demonstrated that automatically generated masks can provide useful, although imperfect, supervision for segmentation model development.

Frozen evaluation on 11,200 Raabin-WBC records revealed reduced performance under dataset shift and identified detector/ROI transfer as an important limitation of end-to-end generalization. Although the proposed framework did not surpass the strongest reference architectures in internal Dice, its contribution lies in integrating weak supervision, detector-guided ROI processing, and explicit end-to-end and external evaluation within a unified framework. These findings support further development of robust localization and improved pseudo-label generation, together with broader multicenter and prospective validation, to strengthen cross-dataset generalization of weakly supervised WBC segmentation.

## Figures and Tables

**Table 1:** The experimental roles of each dataset partition in the study.

Dataset/Subset	No. of Records	Role	Supervision/Reference Used	Model-Development Use	Evaluation Use
Dicle TRAIN	10,305	Fixed internal training partition	Expert-mask-derived bounding boxes for YOLOv13-N detector training	Detector training	No final evaluation
Dicle TRAIN—segmentation-eligible ROIs	10,129	Weakly supervised segmentation training	Automatically generated image-derived pseudo-masks; expert pixel masks not used as segmentation training targets	Proposed segmentation-model training	No final evaluation
Dicle VALIDATION	2208	Internal validation partition	Expert annotations used as reference	Model selection, early stopping, and operating-point selection	Validation only
Dicle TEST	2208	Held-out internal test partition	Expert annotations used as reference	No training, tuning, or model selection	Final internal evaluation
Raabin-WBC	11,200	Independent external test cohort	External reference masks used for evaluation	No training, fine-tuning, threshold selection, or model selection	Frozen external evaluation
Total audited Dicle cohort	14,721	Internal development/evaluation cohort	Blast, Lymphocyte, and Neutrophil records	Partitioned before final model development	Internal evaluation framework

**Table 2:** Audited dataset composition and experimental roles in the study.

Characteristic	Dicle University Dataset	Raabin-WBC Dataset
Study role	Model development and internal evaluation	External evaluation only
Total records used	14,721	11,200
Classes reported	Blast, Lymphocyte, Neutrophil	Global results only; no class-wise reporting
Data split	10,305 TRAIN/2208 VALIDATION/2208 TEST	External test cohort only
Segmentation-training records	10,129 eligible TRAIN ROIs	Not used for training
Image size	256 × 256 pixels	256 × 256 pixels
Annotation/evaluation reference	Expert WBC masks	External reference masks in frozen evaluation cohort
Model selection/tuning	VALIDATION only	None
Final evaluation	Held-out Dicle TEST	Frozen Raabin evaluation

**Table 3:** Performance of the final YOLOv13-N detector on the Dicle validation partition.

Precision	Recall	F1-Score	mAP50	mAP50–95
0.9151	0.8949	0.9049	0.9516	0.7423

**Table 4:** Quality of automatically generated pseudo-masks on the Dicle training verification cohort.

Class	Dice	IoU	Precision	Recall
Blast	0.6528	0.5027	0.8225	0.6001
Lymphocyte	0.8598	0.7674	0.7943	0.9663
Neutrophil	0.5895	0.4399	0.9582	0.4561
Overall	0.7020	0.5717	0.8578	0.6766

**Table 5:** Held-out Dicle test performance of the final proposed framework.

Evaluation Level	Dice	IoU	Precision	Recall
ROI-conditional	0.7188	0.5796	0.8966	0.6422
End-to-end	0.6705	0.5408	0.8378	0.5986

**Table 6:** Ablation analysis of the proposed framework on the held-out Dicle test set.

Configuration	N Paired Records	Mean Dice	ΔDice	95% Bootstrap CI for ΔDice	Wilcoxon W	*p*-Value
Segmentation-only	2063	0.7263	Reference	—	—	—
Full diffusion-assisted model	2063	0.7188	−0.0075	[−0.0083, −0.0066]	533,839	3.61 × 10^−81^

ΔDice denotes the mean paired Dice difference relative to the segmentation-only configuration.

**Table 7:** Comparison of segmentation performance on the held-out Dicle test set.

Method	Evaluation Level	Dice	IoU	Precision	Recall
U-Net	-	0.8047	0.7175	0.8016	0.8556
Attention U-Net	-	0.8053	0.7206	0.8011	0.8562
TransUNet	-	0.8566	0.7961	0.8796	0.8576
Swin-Unet	-	0.8155	0.7384	0.8376	0.8490
MedSegDiff-style	-	0.6531	0.5305	0.6251	0.7847
Proposed framework	ROI-conditional	0.7188	0.5796	0.8966	0.6422
Proposed framework	End-to-end	0.6705	0.5408	0.8378	0.5986

## Data Availability

The Dicle University clinical dataset used in this study contains protected health information and is not publicly available due to privacy and institutional restrictions. De-identified data may be made available to qualified researchers upon reasonable request to the corresponding author, subject to institutional review board approval and a data use agreement. The Raabin-WBC dataset is publicly available.
